# Supplementary material for: Castration Induces Down-Regulation of A-Type K+ Channel in Rat Vas Deferens Smooth Muscle
Source: Int J Mol Sci. 2019 Aug 21;20(17):4073. doi: 10.3390/ijms20174073 (PMC6747096; doi:10.3390/ijms20174073)
Supplement: Supplementary file 1 [file ijms-20-04073-s001.zip › ijms-554645-SI.pdf]

Supplementary Materials

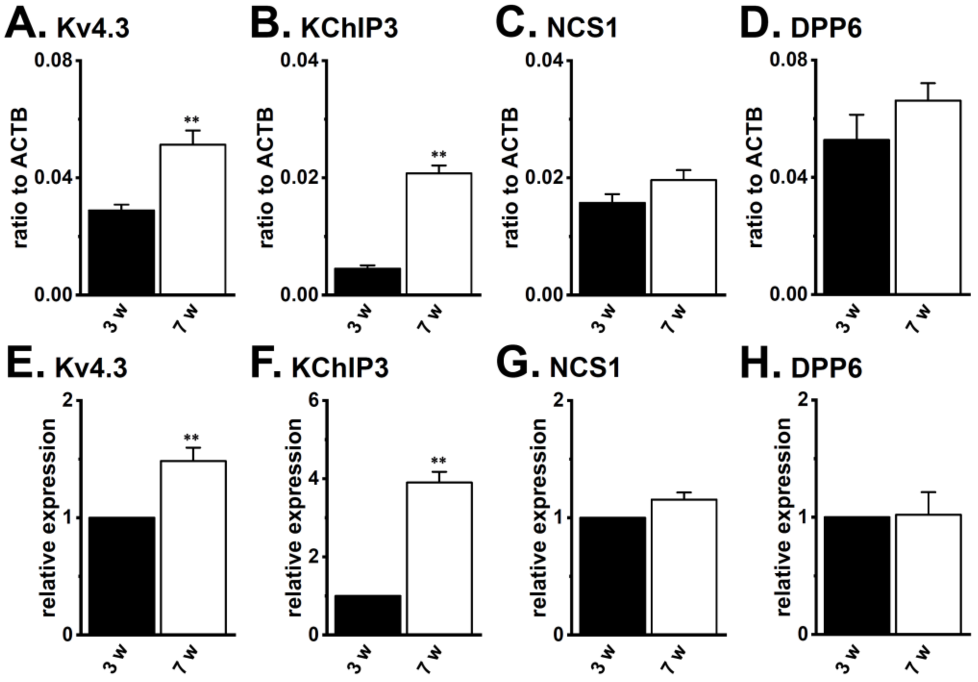

Supplementary Figure S1. Developmental changes in Kv4.3 and KChIP3 expression levels. A-D: The expression of Kv4.3, KChIP3, NCS1, and DPP6-S transcripts in the VDSMs of 3- and 7-week-old rats was examined using a real-time PCR assay. Values are shown for steady-state transcripts relative to ACTB in the same preparation. A: Kv4.3, B: KChIP3, C: NCS1, D: DPP6-S ($n = 6$ for each). **: $p < 0.01$ vs. 3-week-old. E-H: The expression of Kv4.3, KChIP3, NCS1, and DPP6-S proteins in the VDSMs of 3- and 7-week-old rats was examined by Western blotting. Values were expressed as a ratio versus those in 3-week-old rats. E: Kv4.3, F: KChIP3, G: NCS1, H: DPP6-S ($n = 4$ for each). The significance of differences between two groups was evaluated by the Student's t-test, after the F test. **: $p < 0.01$ vs. 3-week-old.
